# Pain control and neonatal outcomes in 211 women under epidural anesthesia during childbirth at high altitude in Qinghai, China

**DOI:** 10.3389/fmed.2024.1361777

**Published:** 2024-04-25

**Authors:** Pengxia Wang, Kaihui Li, Dongliang Wu, Sen Cheng, Yinying Zeng, Peng Gao, Zhibing Wang, Shanshan Liu

**Affiliations:** ^1^Department of Anesthesiology, Affiliated Chenggong Hospital of Xiamen University, Xiamen, China; ^2^Department of Medical Service, Affiliated Chenggong Hospital of Xiamen University, Xiamen, Fujian, China

**Keywords:** labor, epidural, analgesia, altitude, Apgar score

## Abstract

**Background:**

High altitudes are characterized by low-pressure oxygen deprivation. This is further exacerbated with increasing altitude. High altitudes can be associated with reduced oxygenation, which in turn, can affect labor, as well as maternal and fetal outcomes. Epidural anesthesia can significantly relieve labor pain. This study aimed to assess the effects of elevation gradient changes at high altitude on the analgesic effect of epidural anesthesia, labor duration, and neonatal outcomes.

**Methods:**

We divided 211 women who received epidural anesthesia into groups according to varying elevation of their residence (76 in Xining City, mean altitude 2,200 m; 63 in Haibei Prefecture, mean altitude 3,655 m; and 72 in Yushu Prefecture, mean altitude 4,493 m). The analgesic effect was assessed using a visual analog scale (VAS). Labor duration was objectively recorded. The neonatal outcome was assessed using Apgar scores and fetal umbilical artery blood pH.

**Results:**

VAS scores among the three groups did not differ significantly (*p* > 0.05). The neonatal Apgar scores in descending order were: Xining group > Haibei group > Yushu group (*p* < 0.05). The stage of labor was similar among the three groups (*p* > 0.05). Fetal umbilical artery blood pH in descending order were: Xining group > Haibei group > Yushu group (*p* < 0.05).

**Conclusion:**

Elevation gradient changes in highland areas did not affect the efficacy of epidural anesthesia or labor duration. However, neonatal outcomes were affected.

## 1 Introduction

High altitudes are characterized by low-pressure oxygen deprivation, which is further exacerbated with increasing altitude (1). Several recent studies have shown that a series of metabolic and physiological functional changes caused by exposure to high altitudes can negatively affect human organ systems, thus, affecting the health of people working and living in high-altitude areas (2, 3). Numerous studies have shown that prolonged exposure to low-pressure hypoxia causes increased respiratory rate and tidal volume. During acute high-altitude exposures, hypocapnia is the main feature of the hypoxic ventilatory response, which increases blood pH. However, the effect of increased blood pH on pharmacokinetics is unknown and may depend on drug-specific properties (4). High altitude can adversely affect the cardiovascular system, leading to numerous unfavorable outcomes, including an increased risk of arrhythmias, systemic hypertension, high-altitude pulmonary hypertension, right ventricular hypertrophy, and right ventricular failure (5). In a low-oxygen plateau, the gray matter, white matter, arteries, and veins of the brain are altered, which in turn, affects brain functions, especially cognitive function, and induces the development of plateau diseases (6). People living at high altitudes for prolonged periods have poorer renal function and a reduced glomerular filtration rate (GFR) (7). Physiological changes due to altitude stress may affect drug absorption, distribution, metabolism, and excretion, as well as alter drug pharmacokinetics. As such, dose regimens to ensure drug efficacy and safety may need to be altered (7).

Qinghai Province is located in the northeastern part of the Tibetan Plateau, with an average elevation of more than 3,000 m above sea level. High altitude affects hemoglobin levels and is associated with reduced oxygenation, which can affect labor, as well as maternal and fetal outcomes. Labor pain is one of the most excruciating types of pain experienced by a woman (8). Only 9% of mothers can tolerate labor pain (9). Epidural anesthesia effectively relieves labor pain (10, 11). Most studies on epidural anesthesia for labor analgesia have been conducted in areas of normal altitude, e.g., plains. Few studies have been conducted in high-altitude areas, although higher altitudes are associated with greater adverse effects on the human body. Therefore, this study aimed to explore the differences in pain relief and neonatal prognosis after epidural anesthesia for labor analgesia in women who have been living at three different altitudes in the Qinghai region for prolonged periods.

## 2 Patients and methods

### 2.1 Ethics statement

This study was approved by the Ethics Committee of the local hospital (approval number: KY-2021-33). All women in labor signed an informed consent form for epidural anesthesia for labor analgesia. All procedures were conducted in line with the Declaration of Helsinki.

### 2.2 Participants

**Figure 1:**
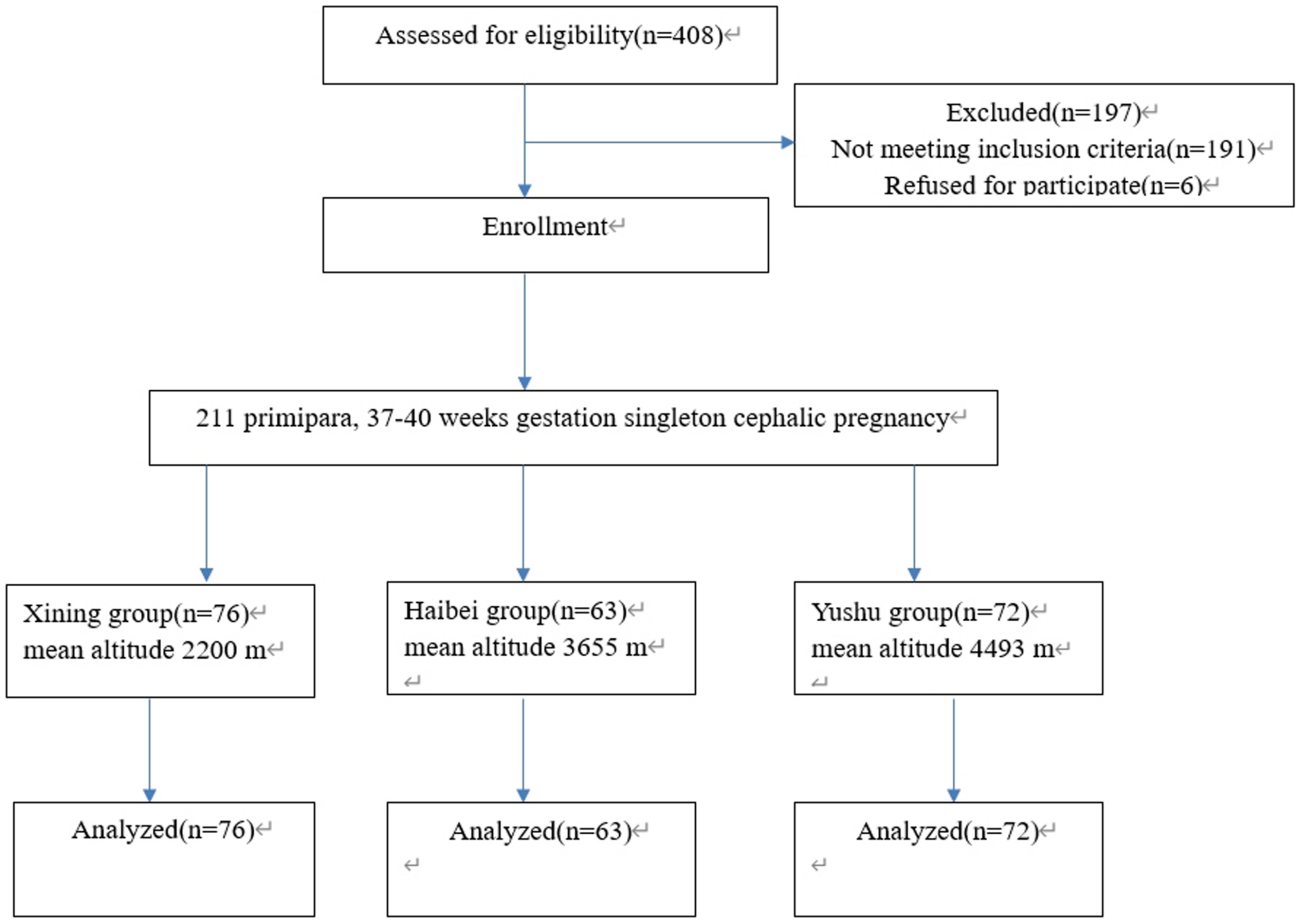
CONSORT flow diagram of study enrollment and analysis.

**Figure 2:**
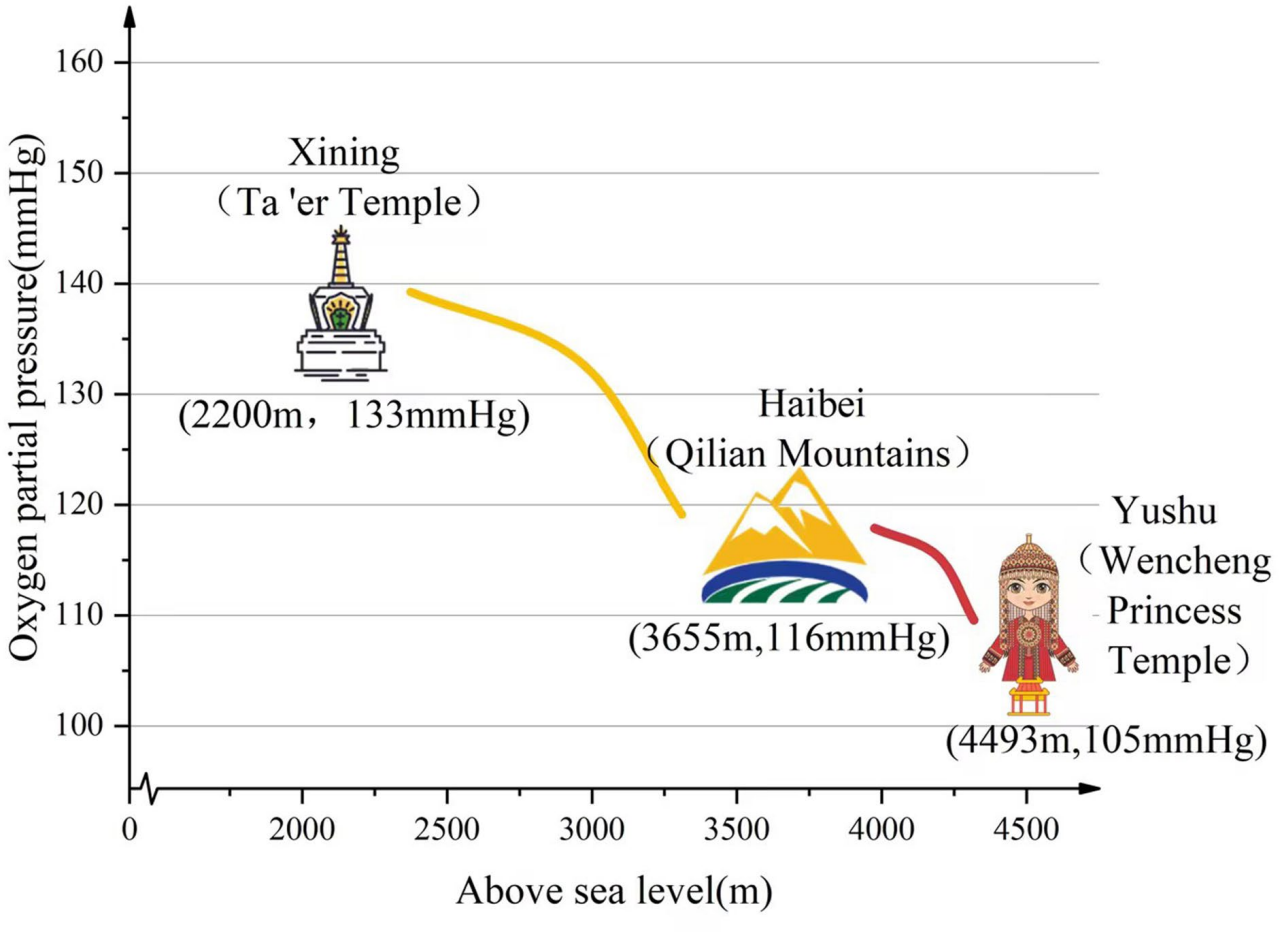
Oxygen partial pressure at different altitudes.

This observational cohort study was conducted at Qinghai Red Cross Hospital (Xining, Chengzhong, China) between October 2020 and October 2021. The study included 211 permanent residents of the Qinghai Province. A total of 408 women were screened for eligibility. Of these, 197 were excluded because they did not meet the inclusion criteria or refused to participate. Finally, 211 completed the study. The 211 women were divided into three groups according to varying altitudes (76 in the Xining group, mean altitude, 2200 m; 63 in the Haibei group, mean altitude, 3,655 m; and 72 in the Yushu group, mean altitude, 4,493 m). All 211 women voluntarily received epidural anesthesia for labor analgesia. This study followed strict inclusion and exclusion criteria. The inclusion criteria were as follows: 23–35 years of age, first vaginal delivery, singleton cephalic position, and 37–40 weeks of gestation. The exclusion criteria were serious medical or surgical illness, mental illness, infection at the puncture site, uncooperative labor, having already received other analgesics, and not a permanent resident of Xining City, Haibei Prefecture, or Yushu Prefecture. Figures 1, 2 show the study flowchart.

### 2.3 Anesthesia management

The mother and her family received epidural anesthesia for labor analgesia voluntarily. The anesthesiologist and obstetrician evaluated and confirmed that epidural anesthesia was not contraindicated. The family provided informed consent for the anesthesia, and the midwife provided intravenous access and connected all the monitoring equipment. The L3–4 space was selected and punctured using a 16-G Tuohy needle when the cervical opening was 0–3 cm. An epidural catheter was inserted 4 cm into the L3–4 space. A test dose of 4 mL of 2% lidocaine hydrochloride was injected into the epidural catheter. After confirming the absence of errors, a patient-controlled epidural analgesic pump was inserted. The formulation consisted of 0.67 mg/mL ropivacaine, 0.33 μg/mL sufentanil, and 138 mL physiological saline solution (total = 150 mL). The analgesic pump parameters were as follows: total volume, 150 mL; first dose, 10–15 mL/time; continuous infusion volume, 8–12 mL/h; self-controlled dosage, 0–12 mL/h; locking time, 40 min; and maximum limit, 30 mL/h.

### 2.4 Information recording

Maternal demographic information included age, ethnicity, gestational week, body mass index, educational background, place of residence (urban/rural), attendance at maternity school, signs of basal vitality (heart rate, blood pressure, and fetal heart rate), and the main indicator (visual analog scale (VAS) scores). The VAS is usually measured using a 10-cm straight line, ranging from 0 cm (no pain at all) to 10 cm (the worst pain imaginable). In this study, the following VAS cut-offs were used: 0–3 cm for mild pain, 4–6 cm for moderate pain, and 7–9 cm for severe pain. Maternal VAS scores were recorded at T0 (5 min before analgesia), T1 (10 min after labor analgesia), T2 (60 min after labor analgesia), T3 (at the opening of the uterus to 10 cm), and T4 (at delivery).

Asphyxia was assessed via five signs: heart rate, respiration, muscle tone, laryngeal reflexes, and skin color within 1 min, 5 min, and 10 min after birth. Each sign was scored between 0–2 points. Heart rate: heart rate greater than 100 beats per minute, 2 points; less than 100 beats per minute, 1 point; no heart rate, 0 points. Breathing: 2 points for even breathing and loud crying; 1 point for slow and irregular breathing or weak crying; and 0 points for no breathing. Muscle tone of limbs: 2 points if limbs are active, 1 point if limbs are slightly flexed, and 0 points if limbs are flaccid. Response to stimuli: After playing with the soles of the feet or inserting a nasal cannula, the baby cries, sneezes, or coughs for two points; only frowns and other slight reactions for one point; and no reaction for zero points. Skin color: Pink skin on the whole body was scored 2 points, pink skin on the trunk, bruising on the limbs was scored 1 point, and bruising or pallor on the whole body was scored 0 points. The total score was calculated as the sum of the scores for the 5 signs (maximum = 10). Labor and neonatal umbilical artery blood pH, which was used to assess for intrauterine hypoxia and neonatal prognosis, were considered as secondary indicators.

The maternal VAS scores, heart rate, blood pressure, and fetal heart rate, were recorded for analysis.

### 2.5 Sample size estimation and blinding

We calculated the sample size with consideration for the primary outcome (adequate analgesia). This estimation was based on an 80% power of the study and a 95% confidence interval. In total, 265 patients were considered adequate.

The nurse anesthesiologist who prepared the drug was aware of the grouping information. However, the anesthesiologist who administered the drug, the patients, and the data collector were blinded to the allocation.

### 2.6 Statistical analysis

The SPSS, version 22.0 (IBM, Chicago, IL, United States) was used for all data analyses. Numerical data are presented as (*x* ± s). The differences among the three groups were assessed using the *t*-test. Comparisons between different time points within the same group were assessed using a paired *t*-test. Categorical data are presented as *n* (%). Comparisons between the groups were performed using the chi-square test. Factors of variables were controlled for with a skewed Analysis of covariance. *p* < 0.05 was considered statistically significant in this study.

## 3 Results

### 3.1 Patient demographics and clinical characteristics

The demographic characteristics of the three groups were comparable in terms of parturition age (years), ethnicity (Han Chinese/ethnic minority), gestational age (weeks), maternal body mass index (kg/m^2^), maternal educational background (secondary/specialized/undergraduate and above), place of residence (urban/rural), participation in maternity schools (yes/no), and newborn weight (g). Table 1 presents the results of this study. Ethnic minorities accounted for a larger proportion of women who gave birth in the Yushu group. In contrast, the urban population predominated among women who gave birth in the Xining group and the number of participants who attended antenatal clinics was also higher (*p* < 0.001). The baseline characteristics of the mothers in the three groups are shown in Table 2. The three groups did not differ significantly in heart rate and blood pressure at each time point. At T1, the Yushu group had the lowest fetal heart rate values, the Haibei group had the second highest, and the Xining group had the highest (*p* < 0.001).

**Table 1:** Demographics of the three groups.

	Xining (*n* = 76)	Haibei (*n* = 63)	Yushu (*n* = 72)	X^2^/*F*	*p*
Age (years)	26.62 ± 3.04	26.97 ± 2.98	26.86 ± 3.11	0.245	0.783
Nationality (%)				74.753	<0.001
Han	54 (71.1)	21 (33.3)^*^	2 (2.8)^*#^		
Majority	22 (28.9)	42 (66.7)^*^	70 (97.2)^*#^		
Gestational age (weeks)	38.91 ± 1.21	38.87 ± 1.16	38.53 ± 1.37	2.014	0.136
Puerpera BMI (kg/m^2^)	28.85 ± 2.53	28.68 ± 2.89	29.35 ± 2.96	1.070	0.345
Educational background (%)				12.516	0.014
Middle school	18 (23.7)	27 (42.9)	36 (50)^*^		
Three-year college	35 (46.1)	24 (38.1)	25 (34.7)^*^		
Bachelor and above	23 (30.3)	12 (19)	11 (15.3)^*^		
Residency (%)				76.651	<0.001
Cities and towns	63 (82.9)	23 (36.5)^*^	9 (12.5)^*#^		
Countryside	13 (17.1)	40 (63.5)^*^	63 (87.5)^*#^		
Participation in maternity schools (%)				50.339	<0.001
Yes	27 (35.5)	45 (71.4)^*^	65 (90.3)^*#^		
No	49 (64.5)	18 (28.6)^*^	7 (9.7)^*#^		
Newborn weight (g)	3209.58 ± 308.54	3162.83 ± 326.24	3134.69 ± 296.35	1.104	0.333

*p <0.05 vs Xining group; ^#^*p* <0.05 vs Haibei group.

**Table 2:** Comparison of baseline information between the three groups.

	Group	*n*	T0	T1	T2	T3	T4
Rate	Xining	76	81.37 ± 11.54	87.41 ± 8.98^a^	83.24 ± 8.01	80.62 ± 6.97	80.14 ± 8.63^a^
	Haibei	63	81.32 ± 10.5	88.98 ± 9.54^a^	83.30 ± 7.73	80.02 ± 8.23	80.65 ± 8.64^a^
	Yushu	72	81.01 ± 9.93	85.61 ± 9.95^a^	81.81 ± 8.35	80.14 ± 7.61	80.64 ± 8.32^a^
	F		0.062	2.399	0.719	0.681	0.306
	*p*		0.939	0.147	0.489	0.507	0.273
Systolic blood pressure	Xining	76	113.50 ± 8.43	103.54 ± 8.62^a^	110.46 ± 8.49^a^	113.42 ± 10.22	112.64 ± 9.54
	Haibei	63	114.06 ± 9.62	102.90 ± 8.77^a^	110.25 ± 10.19^a^	111.37 ± 8.87	113.71 ± 9.80
	Yushu	72	113.03 ± 8.97	103.44 ± 9.11^a^	110.22 ± 9.03^a^	112.72 ± 8.72	112.12 ± 10.04
	F		0.274	0.084	0.136	0.679	0.845
	*p*		0.761	0.919	0.873	0.508	0.431
Diastolic blood pressure	Xining	76	71.96 ± 6.55	71.50 ± 8.10	71.89 ± 8.21	71.80 ± 6.57	71.55 ± 6.38
	Haibei	63	71.62 ± 6.26	71.83 ± 5.93	71.08 ± 7.23	71.05 ± 7.01	71.60 ± 6.09
	Yushu	72	71.36 ± 7.60	71.17 ± 6.86	71.74 ± 6.49	71.89 ± 7.89	71.53 ± 6.61
	F		0.676	0.830	0.253	0.201	0.057
	*p*		0.510	0.438	0.777	0.818	0.945
Fetal heart	Xining	76	135.76 ± 4.38	127.14 ± 4.83^a^	133.26 ± 4.28^a^	136.45 ± 4.28	135.91 ± 4.55
	Haibei	63	136.46 ± 4.27	125.17 ± 3.90^*a^	133.27 ± 4.69^a^	136.68 ± 4.35	136.35 ± 4.48
	Yushu	72	136.89 ± 3.85	121.39 ± 4.25^*#a^	133.04 ± 4.58^a^	136.46 ± 4.33	135.81 ± 4.02
	F		0.002	10.479	0.025	0.800	0.609
	*p*		0.998	<0.001	0.976	0.451	0.545

*p <0.05 vs Xining group; ^#^*p* <0.05 vs Haibei group; ^a^*p* <0.05 vs T0.

### 3.2 Main indicators

#### 3.2.1 Assessment of analgesic efficacy

**Figure 3:**
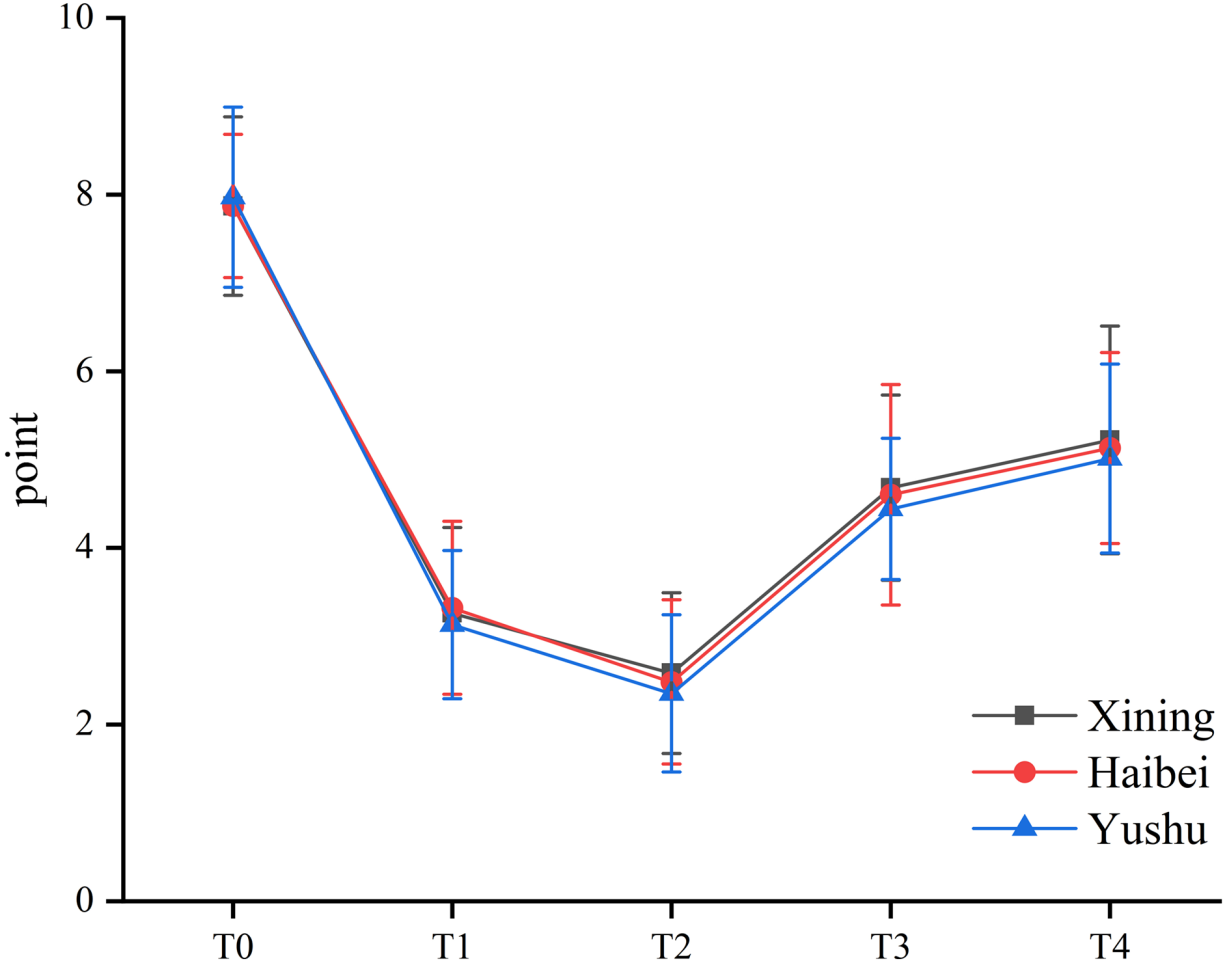
The VAS scores among the three groups.

As shown in Table 3 and Figure 3, the VAS scores among the three groups were similar (*p* > 0.05).

**Table 3:** VAS score associated with Xining vs Haibei vs Yushu.

	Group	*n*	T0	T1	T2	T3	T4
VAS score	Xining	76	7.87 ± 1.01	3.64 ± 1.02^a^	2.82 ± 0.92^a^	4.97 ± 1.03^a^	5.47 ± 1.13^a^
	Haibei	63	7.87 ± 0.81	3.24 ± 1.04^*a^	2.48 ± 0.93^*a^	4.56 ± 1.33^*a^	5.08 ± 1.15^*a^
	Yushu	72	7.97 ± 1.02	2.82 ± 1.09^*#a^	2.13 ± 0.96^*#a^	3.82 ± 1.07^*#a^	4.50 ± 0.99^*#a^
	F		0.085	4.400	12.326	13.216	4.968
	*p*		0.919	0.015	<0.001	<0.001	0.008

*p <0.05 vs Xining group; ^#^*p* <0.05 vs Haibei group; ^a^*p* <0.05 vs T0.

#### 3.2.2 Neonatal outcomes

**Figure 4:**
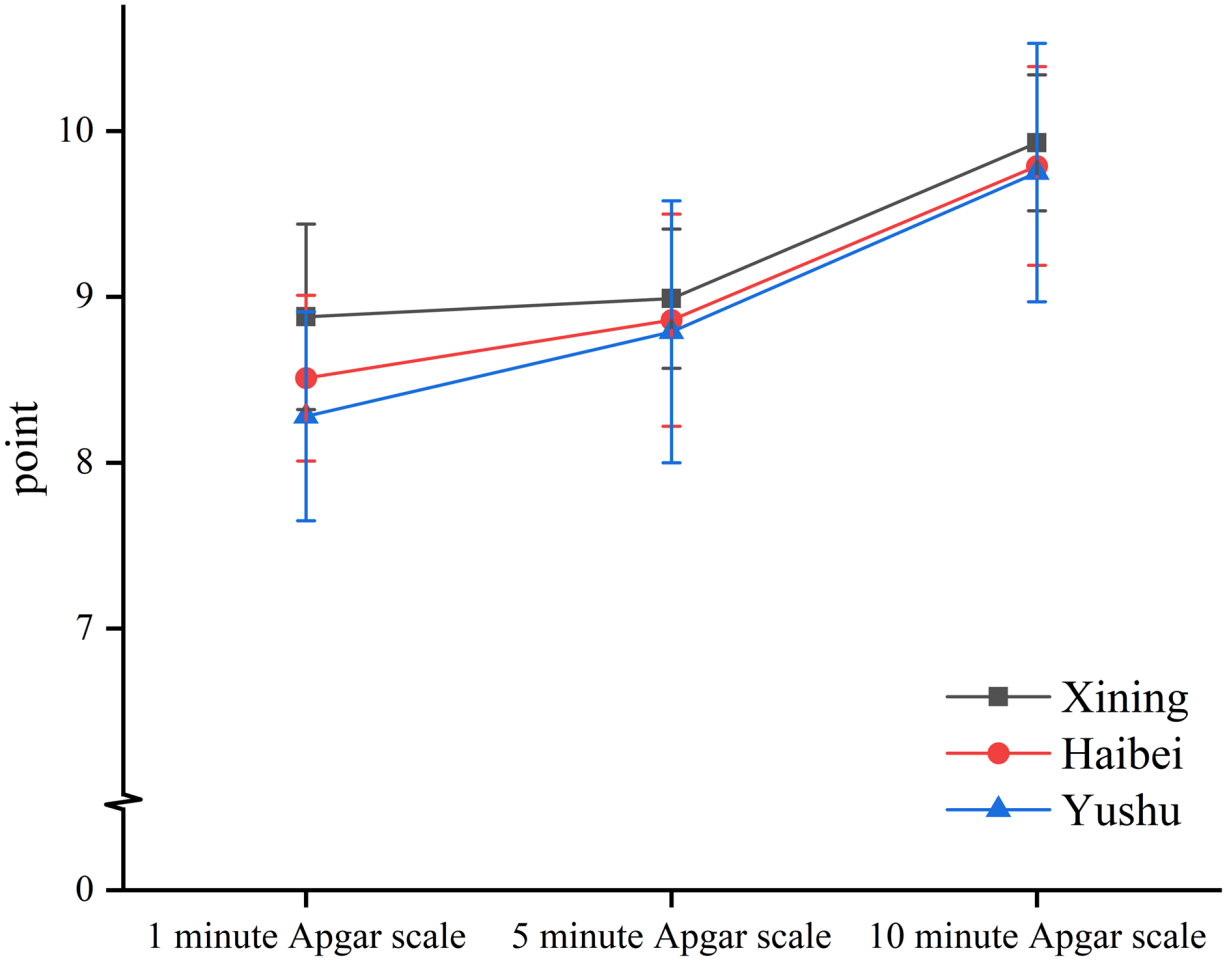
Apgar scores among the three groups.

**Figure 5:**
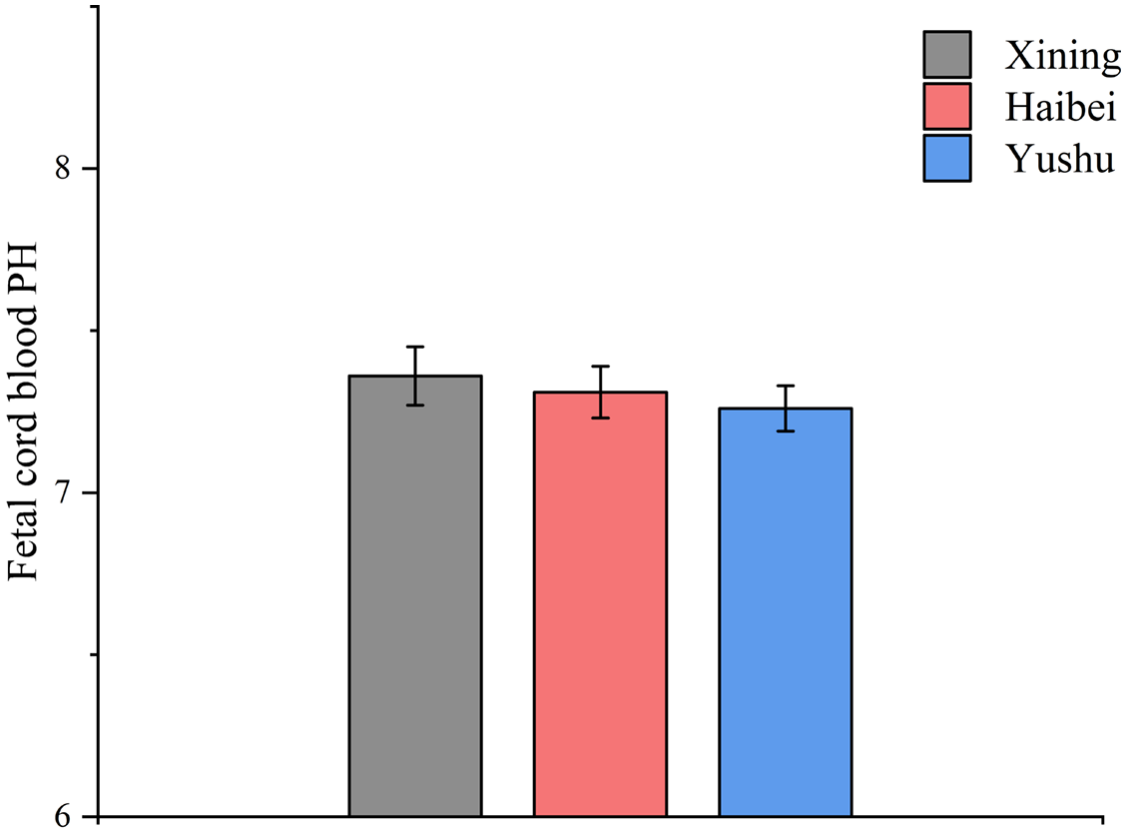
Fetal cord blood PH among the three groups.

The 1-min Apgar score was the highest in the Xining group, followed by the Haibei group, and the lowest in the Yushu group (*p* < 0.001). The three groups did not differ significantly in the 5- and 10-min Apgar scores (*p* > 0.05). As shown in Table 4 and Figures 4, 5, fetal umbilical arterial blood pH was higher in the Xining group, followed by the Haibei group, and the lowest in the Yushu group (*p* < 0.001).

**Table 4:** Comparison of neonatal Apgar scores between the three groups.

	Xining (*n* = 76)	Haibei (*n* = 63)	Yushu (*n* = 72)	X^2^/*F*	*p*
1 min Apgar scale (points)	8.88 ± 0.56	8.51 ± 0.50^*^	8.28 ± 0.63^*#^	7.915	<0.001
5 min Apgar scale (points)	8.99 ± 0.42	8.86 ± 0.64	8.79 ± 0.79	5.287	0.506
10 min Apgar scale (points)	9.93 ± 0.41	9.79 ± 0.60	9.75 ± 0.78	0.649	0.524
Fetal cord blood PH	7.36 ± 0.09	7.31 ± 0.08^*^	7.26 ± 0.07^*#^	11.725	<0.001

*p <0.05 vs Xining group; ^#^*p* <0.05 vs Haibei group.

#### 3.2.3 Labor stage assessment

**Figure 6:**
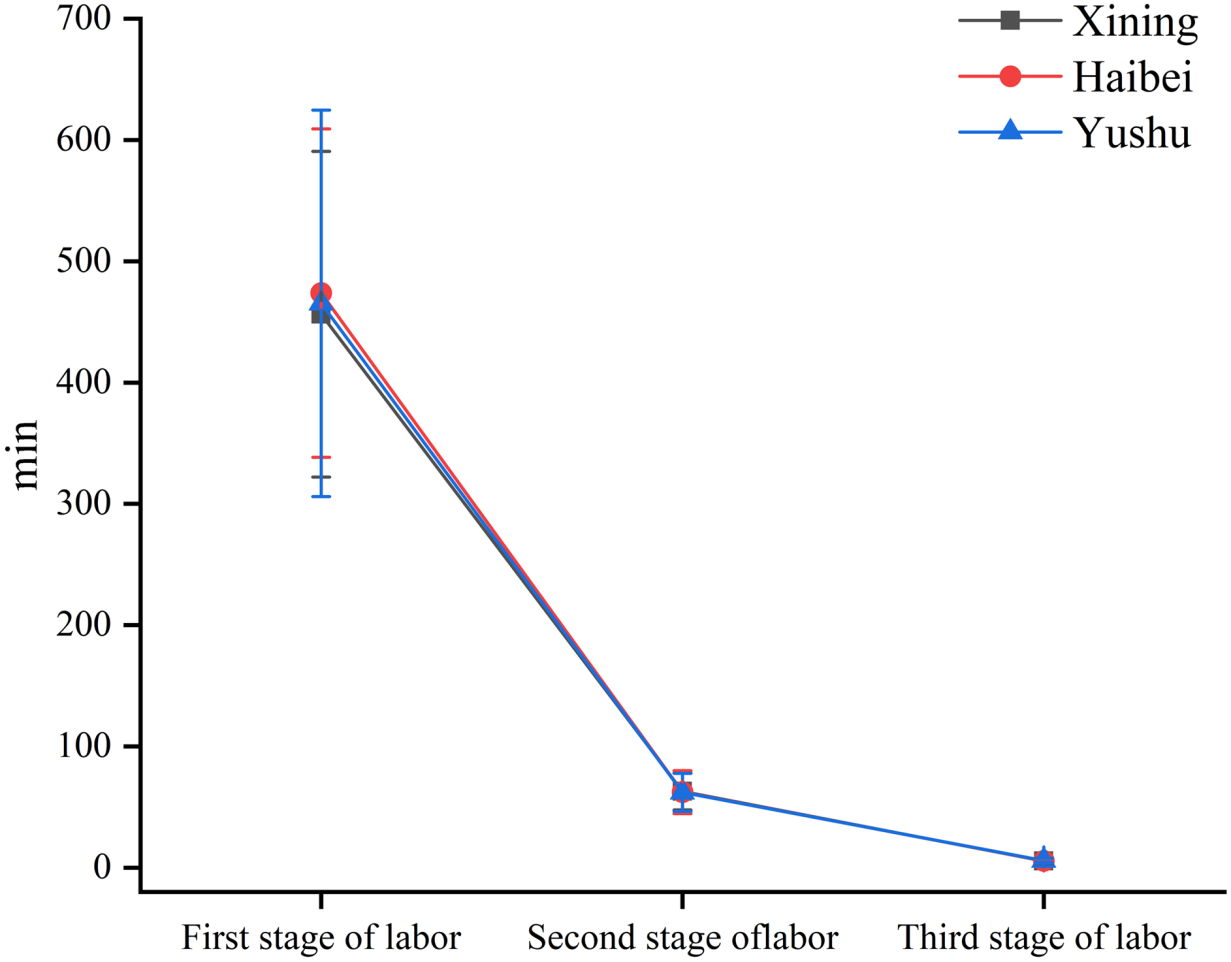
Labor duration among the three groups.

Labor duration did not differ significantly among the three groups (*p* > 0.05, Table 5; Figure 6).

**Table 5:** Stage of labor associated with Xining vs Haibei vs Yushu.

Group	*n*	First stage of labor (min)	Second stage of labor (min)	Third stage of labor (min)
Xining	76	456.34 ± 134.17	62.93 ± 15.36	5.54 ± 2.04
Haibei	63	473.76 ± 135.42	62.33 ± 17.77	5.60 ± 1.80
Yushu	72	465.26 ± 159.3	62.14 ± 15.73	5.83 ± 2.08
F		0.200	0.347	0.149
*p*		0.819	0.707	0.862

## 4 Discussion

This study compared the differences in analgesic effects among women who received epidural anesthesia at different altitudes in a high-altitude region and neonatal prognosis. The three groups of women resided in Qinghai at different altitudes and received the same epidural anesthesia for labor analgesia. Our results suggest that labor at locations of increasing altitude affects neonatal prognosis despite a painless labor. Importantly, a higher altitude resulted in lower 1-min Apgar scores among the neonates and lower neonatal umbilical arterial blood pH, which adversely affects neonatal outcome but demonstrated no effect on analgesic efficacy or labor duration.

The environmental characteristics of low-pressure hypoxia at high altitudes are great threats to human beings. The higher the altitude, the lower the atmospheric pressure and partial pressure of oxygen. At altitudes beyond 2,500 m, the partial pressure of oxygen declines even further, which in turn, leads to an exponential reduction in arterial oxygen saturation (SaO2) (4). In the 1960s, Grahn and Kratchmann reported that atmospheric pressure was significantly correlated to neonatal mortality (12). At high altitudes, pregnant women have less available iron and vitamins A and D, higher erythropoietic requirements, and lower arterial oxygen levels during the antenatal period (13). Adequate oxygen for fetal development depends not only on atmospheric oxygen levels, but also on the efficient transport of oxygenated blood via the uteroplacental circulation and the cardiac output in women also reduces during pregnancy (14–16). Therefore, the efficient transport of oxygenated blood into the uteroplacental circulation is further compromised, affecting fetal development. To quickly assess the clinical status of a newborn within 1 min and determine whether timely intervention is needed to restore respiration, Dr. Virginia Apgar devised a scoring system in 1952 to provide a standardized assessment of the infant after birth (the Apgar score) (17). The Apgar score consists of five indicators, including skin color, heart rate, reflex irritability, activity/flexion, and respiratory effort. Each indicator can be scored on a scale of 0–2 for a summed total of 10 points. A score of 8–10 indicates good condition, 4–7 indicates fair condition, and 0–3 indicates poor condition (18). In a controlled study of newborns in Cerro de Pasco (4,340 m above sea level) and Lima (150 m above sea level), Peru, arterial oxygen saturation and 1-min Apgar scores were lower in newborns who were born at higher altitudes (19). In this study, the Yushu group demonstrated the lowest 1-min Apgar scores, while the Xining group showed the highest. The Haibei group demonstrated 1-min Apgar scores that were between the other two groups. These results demonstrated that the 1-min Apgar scores decreased with increasing altitude. Notably, the 1-min Apgar scores in all three groups were above 8, indicating good neonatal conditions.

In contrast, a study in Colorado, United States, identified lower neonatal birthweights at high altitudes as a potentially significant factor for reduced neonatal outcomes (20). The average weight of neonates in the Yushu group (highest altitude group) was 3 kg. Numerous factors can influence neonatal Apgar scores, including maternal sedation or anesthesia during labor, congenital malformations, gestational age, trauma, and inter-observer variability. The 1-min Apgar score assesses acute or transient neonatal problems. Conversely, the 5-min Apgar score is more appropriate for assessing neonatal prognosis as it reflects measures, such as prior resuscitation, and the score is not related to birthweight (17). If the 5-min Apgar score was low, the score should be measured again at 10 min (21). In the present study, the 5- and 10-min Apgar scores between the three groups did not differ significantly. The lack of difference may be due to a high neonatal 1-min Apgar score in all three groups, and the neonates were provided with oxygen and warmth immediately after birth. Fetal heart decreased after 10 min of analgesia in all three groups of mothers in this study. The early decrease in fetal heart rate may be related to uterine contractions and vagal excitation due to fetal head compression (22).

Cord blood pH and lactate dehydrogenase levels are used to predict fetal hypoxia-related damage and metabolic acidosis (21). A recent study concluded that cord blood pH at birth, assessed in conjunction with Apgar scores, is a good predictor of asphyxia severity at birth and early neonatal prognosis (23). When the products of anaerobic metabolism exceed the buffering capacity of fetal arterial blood, the pH of the umbilical artery decreases because of decreased oxygen availability to body tissues and increased lactate content (24). Prolonged gestation, premature rupture of the membranes, low amniotic fluid, and/or fetal growth retardation can decrease the pH of umbilical cord arterial blood in newborns (25, 26). Transient fetal and placental hypoxia may result urteroplacental perfusion reduces by 60% or more due to uterine contractions during delivery (25). The greatest physiological challenge for women in late pregnancy is maintaining sufficient oxygenated blood to supply the uteroplacental circulation and ensure proper fetal development. Oxygen transport is made more difficult by living in high-altitude and hypoxic environments for prolonged periods. Although physiological adjustments can be made to counteract arterial hypoxemia to promote hemodynamic stability and increase uteroplacental blood flow, these adjustments can negatively affect newborns (27). In this study, the pH of the neonatal umbilical cord arterial blood in the Xining group was the highest, followed by that of the Haibei group. Conversely, the Yushu group had the lowest umbilical cord arterial blood pH. Despite the differences, the pH was above 7.2 in all three groups. Our results confirmed that an increase in altitude can affect the fetal umbilical artery blood.

Some studies have found that maternal hypotension caused by epidural anesthesia for labor analgesia decreases the fetal umbilical cord blood pH (28). In this study, all three groups received epidural anesthesia for labor analgesia. All of our mothers had a transient decrease in blood pressure 5 min after the onset of analgesia, but the maternal blood pressure gradually recovered soon after. Further studies are needed to confirm whether cord blood pH is affected by this phenomenon.

Pain during labor comes primarily from uterine contractions and cervical dilatation. These stimulations are transmitted to the spinal nerves at T10–L1, leading to visceral pain. Descent of the fetal head stretches the perineum and vagina, and pain fibers in the pudendal and spinal nerves in S2–4 are activated (29). Epidural analgesia, which involves injecting opioids and other adjuvants through an epidural catheter into the epidural space, blocks these pathways to achieve analgesia (30). The World Health Organization recognizes this as an effective analgesic strategy for relieving labor pain (31). Physiological changes induced by altitude stress may affect drug absorption, distribution, metabolism, and excretion and alter drug pharmacokinetics (32). However, the present study found no differences in maternal VAS scores among the three groups. Our results may be due to the mothers being adapted to high-altitude living over prolonged periods (6, 33). Genomical adaption may also contribute to this (34).

Some studies have shown that labor duration is related to the number of births, maternal age, newborn weight, medical interventions, and other factors (35). Greenberg et al. found that older maternal ages correlated with longer first stages of labor in both primiparous and transient labor (36). Similarly, Chen et al. reported that older women had prolonged first and second stages of labor (35). Moreover, medical interventions during labor can also prolong the first and second stages of labor (37). Furthermore, recent studies have shown that epidural labor analgesia prolongs the first and second stages of labor (38). On the contrary, Feng et al. found that epidural labor analgesia did not affect the labor duration (39). Our results also showed that epidural labor analgesia did not affect the course of labor, and the course of labor between the three groups did not differ significantly. In this study, the difference in altitude did not affect the labor duration in women who received epidural labor analgesia.

## 5 Limitation

This study has some limitations. We only included women who receive epidural labor analgesia at varying high altitudes and did not include data from the plains for comparison. This conclusion may not apply to other high-altitude regions because of variations in climate and human environment, economic base, racial differences, educational backgrounds, etc. in each region. In the future, studies should include a control group from the plains to enhance the study design. Future studies should also consider including a greater number of women in labor, focusing on the effects of differences in racial, economic, educational, and other indicators on the mothers and newborns living at high altitudes. Furthermore, the different effects between general anesthesia and nerve block anesthesia at high altitudes should also be further investigated.

## 6 Conclusion

Changes in elevation gradients in highland areas do not affect the efficacy of epidural labor analgesia or labor duration, but they do affect neonatal outcomes. Our findings provide a good guide for local physicians in the planning for birth deliveries and maternal and neonatal interventions, especially for expectant mothers living at high altitudes for a long time or when they travel to high altitudes to practice medicine. Doctors should also inform women of reproductive age who want to move to high altitudes about the need for health assessment for themselves and their children.

## Data availability statement

The raw data supporting the conclusions of this article will be made available by the authors, without undue reservation.

## Ethics statement

The studies involving humans were approved by Ethics Committee of Qinghai Red Cross Hospital. The studies were conducted in accordance with the local legislation and institutional requirements. The participants provided their written informed consent to participate in this study. Written informed consent was obtained from the individual(s) for the publication of any potentially identifiable images or data included in this article.

## Author contributions

PW: Conceptualization, Data curation, Formal analysis, Investigation, Methodology, Resources, Software, Supervision, Writing – original draft, Writing – review & editing. KL: Investigation, Methodology, Writing – review & editing. DW: Writing – review & editing. SC: Data curation, Writing – review & editing. YZ: Software, Writing – review & editing. PG: Supervision, Writing – review & editing. ZW: Data curation, Resources, Investigation, Project administration. SL: Project administration, Supervision, Validation, Visualization, Writing – original draft, Writing – review & editing.
